# Prevalence and surveillance of tuberculosis in Yemen from 2006 to 2018

**DOI:** 10.1017/S0950268822001261

**Published:** 2022-07-20

**Authors:** Wadee Abdullah Al-Shehari, Yi-An Yin, Xinyang Wang, Ying Wang, Haobo Sun, Yingmei Fu, Fengmin Zhang

**Affiliations:** 1 Department of Microbiology, Basic Medical Sciences College, Harbin Medical University, Harbin 150086, China; 2 Department of Medical Microbiology, Faculty of Sciences, Ibb University, Ibb, Yemen; 3 Wu Lien-Teh Institute, Harbin Medical University, Harbin 150086, China

**Keywords:** Concomitant diseases, incidence, tuberculosis, Yemen

## Abstract

Tuberculosis is a major public health issue in Yemen, a country located at the southwestern tip of the Arabian Peninsula, while the situation of tuberculosis had been further exacerbated since the war started in 2015. The objective of this study is to investigate the incidence of tuberculosis in Yemen before the outbreak of COVID-19, from 2006 to 2018. During the 13-year period, 92 482 patients were enrolled in the TB programme records from the 22 governorates. Almost equal number of cases were diagnosed between males and females (a male to female ratio, 1.03:1). A notable rising incidence was observed in all age groups starting from 2011. The sharpest increase occurred in children under age 15, rising by 8.0-fold from 0.5 in the period 2006–2010 to 4.1 in the period 2011–2018. Paediatric TB accounted for 9.6% of all reported cases. In terms of the patient residence, incidence has more than doubled in Sana'a city, Sana'a Gov., Hajjah and Saadah. Concomitant diseases with tuberculosis included diabetes mellitus (14.0%), brucellosis (6.1%), hepatitis (6.0%), rheumatoid arthritis (4.3%), renal disorders (2.5%) and HIV infection (2.5%). Development of interventions to reduce tuberculosis incidence in children and concomitant communicable diseases is urgently needed.

## Introduction

Although the incidence of tuberculosis (TB) has been slowly decreasing worldwide, the global disease burden remains high. In 2020, an estimated 10 million people fell ill with TB worldwide, with number of people who die from TB increasing to an estimated 1.5 million [1]. The COVID-19 pandemic has reversed years of progress in reducing the TB deaths, nearly returning to the level of 2017 [1, 2]. It is predicted that TB will rank as the second leading cause of death from a single infectious agent, after COVID-19 [3].

TB is considered one of the major infectious diseases in the list of Yemen National Disease. The major factors accounting for the high incidence of TB in the country comprise the rapidly growing population, weak health services, the extremely low annual income and the poor social-economic situation in the whole country. This study aimed to determine the incidence of TB with association with geographical locations and concomitant diseases within 13-years, from 2006 and ending in 2018, before the outbreak of COVID-19.

## Materials and methods

### Data sources

**Fig. 1.**
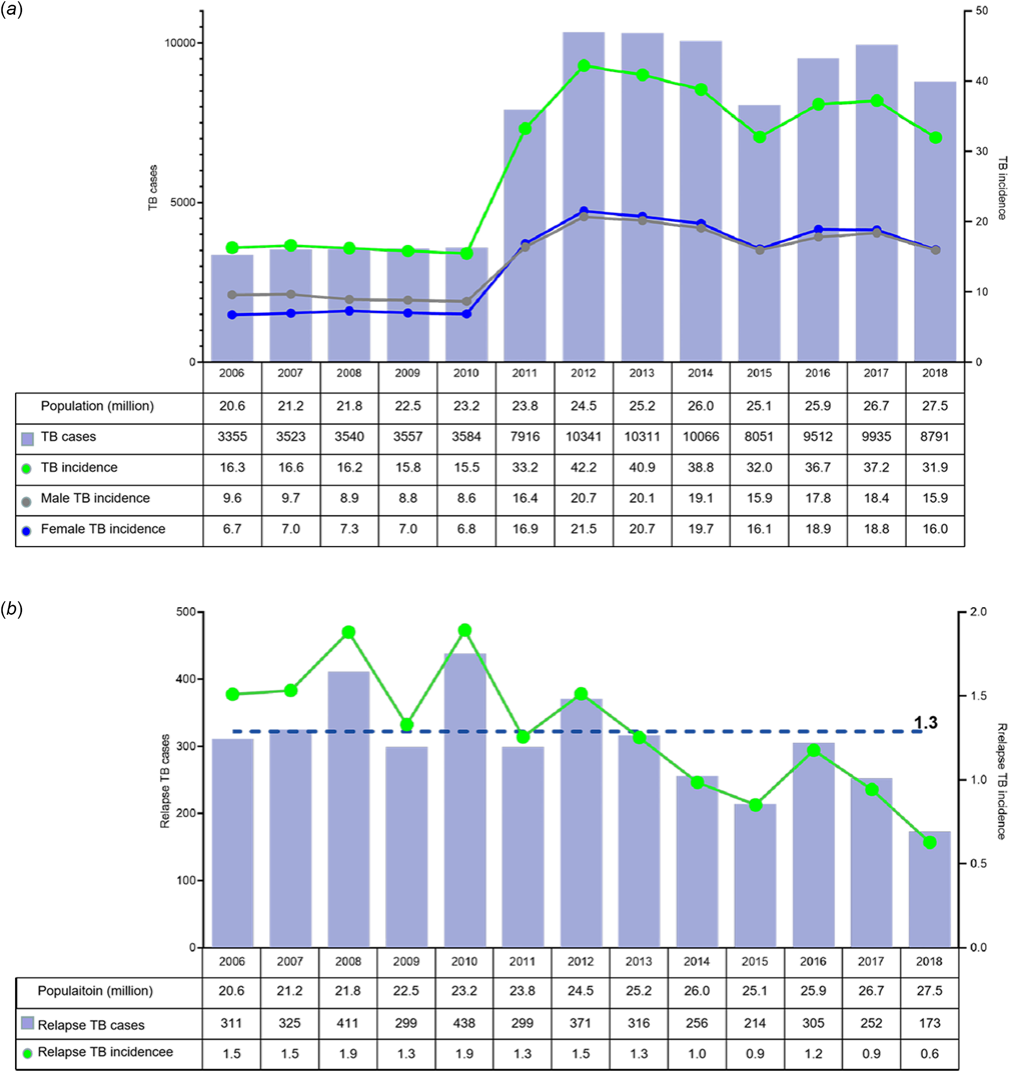
The numbers and incidence (per 100 000 population) of tuberculosis cases in Yemen, 2006–2018. (a) The total cases and incidence of TB. (b) The relapse cases and incidence of TB, dashed line indicates the mean relapse rate (1.3 per 100 000 population) during the 13-year period.

Yemen TB centre released the demographic data related to the occurrence of TB. Permission of the data retrieval and analysis was authorised to this study by National TB Control Programme, Ministry of Health & Population, Republic of Yemen. From 2019, data are impossible to obtain due to the outbreak of COVID-19. Therefore, data from 2006 to 2018 were included in the present study, which involve a total of 92 482 cases reported in TB programme records. Verified cases included both laboratory-confirmed TB and clinically diagnosed TB without laboratory confirmation. We captured records that included the following demographic, clinical and microbiological data: age, sex, residence, diagnostic centre, referral source, presentation date, whether newly or relapse status, initial and periodic sputum microscopy tests, and concomitant diseases. The annual incidence of TB is shown in Figure 1.

### Data analysis

A descriptive analysis of the selected variables was performed using the Epi version 7.0 computer software (CDC Atlanta). Frequency distribution of different variables and percentages of cases were calculated. TB was correlated with residence location by calculating the odds ratio, confidence interval and significance by the calculated *χ*^2^ and the *P* value. *P* < 0.05 was considered statistically significant.

## Results

### Overall characteristics of TB incidence

During the 13-year study period, 92 482 patients were enrolled in TB programme records. In the total reported cases, 64.7% (59 858/92 482) were smear positive; 58.8% (54 355/92 482) were pulmonary TB; 35.5% (32 877/92 482) were associated with concomitant diseases, including diabetes mellitus (DM), brucellosis, viral hepatitis, rheumatoid arthritis, renal disorders, HIV, leukaemia, systemic lupus erythematosus (SLE). In addition, 3970 patients were relapse TB.

There has been an increasing trend of TB incidence over recent years (Fig. 1a). The incidence in 2006 was 16.3/100 000 (3355/20 589 553), which was relatively low and remained stable until 2010. The incidence increased significantly from 2011 with the number of reported cases rising sharply to 33.2/100 000 (7916/23 832 347) and reached 42.2/100 000 (10 341/24 526 211) in 2012. The incidence maintained a high level from then on (Fig. 1a). There were slight decreases in 2015 and 2018, with the number of patients being 8051 and 8791, respectively. These results demonstrated that TB incidents have increased significantly during the years of 2011–2018 compared to that in the period of 2006–2010 (Fig. 1a).

The trends in incidence rates of men and women are basically consistent with the trend in overall incidence rates of population. In 2010, the incidence rate for men and women was 8.6 and 6.8 per 100 000, respectively. In 2011, the incidence rates increased sharply, for male being 16.4 and female being 16.9 per 100 000. It maintained a relatively high level from 2011 to 2018, with a slight decrease in 2015, which in male being 15.9 and in female being 16.1 per 100 000 persons (Fig. 1a).

During the 13-year period, relapse rates from 2006 to 2010 were significantly high, compared to the overall mean relapse rate (1.3 per 100 000), while relapse incidence rates in the years 2013–2018 actually decreased significantly (Fig. 1b).

### Trends of TB preference: age and sex

**Fig. 2.**
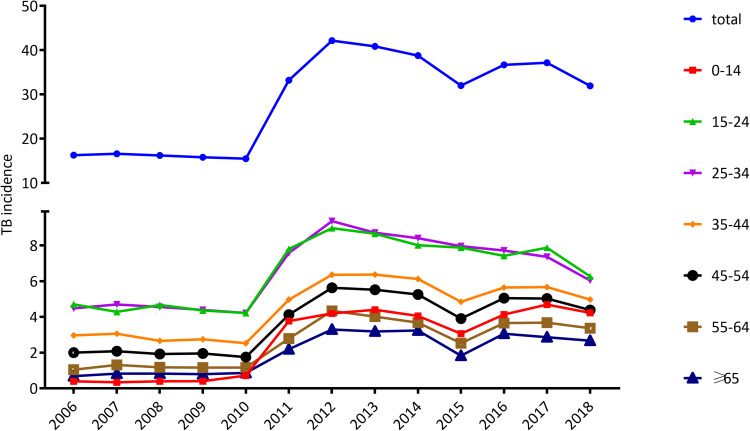
Trends in the incidence of tuberculosis (per 100 000 population) by age.

As shown in Figure 2, in 2006, the incidence rates of 15–24, 25–34, 35–44, 45–54, 55–64, ≥65 and 0–14 age groups were 4.7, 4.5, 3.0, 2.0, 1.0, 0.7 and 0.4 per 100 000 population, respectively, decreasing in turn. Afterward, a stable trend was observed till 2010 and rose sharply from 2011 to 7.8, 7.6, 5.0, 4.1, 2.8, 2.2 and 3.8 per 100 000 population in each of the above age groups. A slight decline in incidence was in 2015 in ages of 35–44, 45–54, 55–64, ≥65 and 0–14, being 4.8, 3.9, 2.5, 1.8 and 3.1 per 100 000 population respectively. The trend in incidence rates of each age group is basically consistent with the trend in the overall incidence (Fig. 2).

Table 1 listed the number and incidence of cases in each age group. When the differences between the TB incidence in the period 2006–2010 to 2011–2018 were examined, a notably age-specific increase could be found (Table 1). The incidence of paediatric TB (TB in children under 15 years of age) jumped the most dramatically from 0.5 in the period 2006–2010 to 4.1 in the period from 2011 to 2018, increasing by 8.0-fold. Children under ages 15 represent for 2.8% of the TB cases in 2006–2010 and the proportion rise to 11.1% in 2011–2018, increasing by 4.0 times. Paediatric TB account for 9.6% of all cases during the 13-year period. The second fastest increasing was seen in the elderly people (>65 years), whose incidence and proportion rise by 2.5 and 1.5 times, respectively (Table 1).

**Table 1.** The differences between the number of TB cases in the period 2006–2010 to 2011–2018 in different age groups

Age groups	Number of cases (%)	Mean incidence			Mean proportion^a^		
		2006–2010	2011–2018	Increase fold^b^	2006–2010	2011–2018	Increase fold^b^
0–14	8847 (9.6%)	0.5	4.1	8	2.8	11.1	4.0
15–24	20 915 (22.6%)	4.4	7.9	0.8	27.7	21.4	0.8
25–34	20 997 (22.7%)	4.5	7.9	0.8	27.8	21.5	0.8
35–44	14 558 (15.7%)	2.8	5.6	1	17.3	15.4	0.9
45–54	12 082 (13.1%)	1.9	4.9	1.5	12.1	13.3	1.1
54–65	8475 (9.2%)	1.2	3.5	2	7.3	9.6	1.3
>65	6608 (7.1%)	0.8	2.8	2.5	5	7.6	1.5
Total	92 482	16.1	36.6	1.3	100	100	

a Indicating the proportion of number in the cases divided by the total number of tuberculosis cases in each age group (*N* = 92 482).

b Increase fold from 2006–2010 to 2011–2018.

**Fig. 3.**
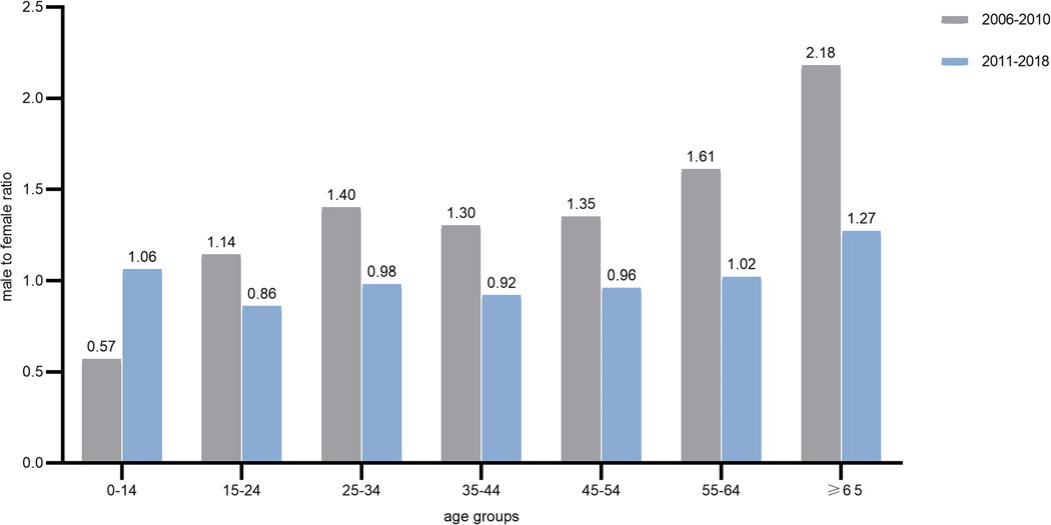
Gender ratio of tuberculosis in different age groups from 2006 to 2018.

From 2006 to 2018, almost equal number of TB cases were notified in male and female, with a pooled male to female ratio 1.03:1. As shown in Figure 3, before the year of 2011, a higher male TB incidence was observed in groups over 15 years old. In contrast, except for the elderly people over 65 years, sex difference in the incidence was vanished in most age groups since 2011.

### Trends of TB preference: governorates

When the residence governorate was considered, there was a high probability of occurrence of TB in Aden (OR 3.2, 95% CI 3.16–3.3, *P* < 0.0001), followed by Al-Hodeidah (OR 1.48, 95% CI 1.46–1.51, *P* < 0.0001); followed by Hajjah (OR 1.5, 95% CI 1.48–1.55, *P* < 0.0001) (Table 2).

**Table 2.** Socio-demographic characteristics of tuberculosis patients enrolled in Yemen 2006–2018: infection rates and risk of association with tuberculosis

Governorate	Number of cases (proportion %)	OR	95% CI	*χ*^2^	*P*
Abyan	2249 (2.4)	1.09	1.05–1.14	18	<0.0001
Aden	8576 (9.3)	3.2	3.16–3.3	1921	<0.0001
Al-Baidah	1755 (1.9)	0.64	0.61–0.67	347	<0.0001
Al-Dahleh	1435 (1.6)	0.64	0.60–0.67	284	<0.0001
Al-Hodeidah	14 409 (15.6)	1.48	1.46–1.51	1937	<0.0001
Al-Jawf	1802 (1.95)	0.85	0.82–0.9	36	<0.0001
Al-Mahrah	558 (0.6)	1.32	1.2–1.44	446	<0.0001
Al-Mahweet	1178 (1.3)	0.49	0.47–0.52	85	<0.0001
Al-Mukalla	1815 (1.96)	0.82	0.78–0.89	69	<0.0001
Amran	2591 (2.8)	0.61	0.59–0.63	607	<0.0001
Dhamar	5260 (5.7)	0.83	0.80–0.85	168	<0.0001
Hajjah	10 155 (11)	1.5	1.48–1.55	1603	<0.0001
Ibb	6421 (6.9)	0.61	0.60–0.63	1430	<0.0001
Laheg	3738 (4.1)	1.1	1.0–1.13	32	<0.0001
Mareb	1304 (1.4)	1.16	1.1–1.23	29	<0.0001
Raimah	604 (0.65)	0.32	0.29–0.30	859	<0.0001
Saadah	1765 (1.9)	0.53	0.50–0.55	719	<0.0001
Sana'a city	10 822 (11.7)	1.33	1.3–1.35	774	<0.0001
Sana'a Gov.	2529 (2.7)	0.57	0.54–0.59	785	<0.0001
Sayoun	713 (0.77)	0.25	0.24–027	1495	<0.0001
Shabwah	964 (1.04)	0.42	0.40–0.45	726	<0.0001
Taiz	8867 (9.6)	0.76	0.74–0.78	575	<0.0001

**Fig. 4.**
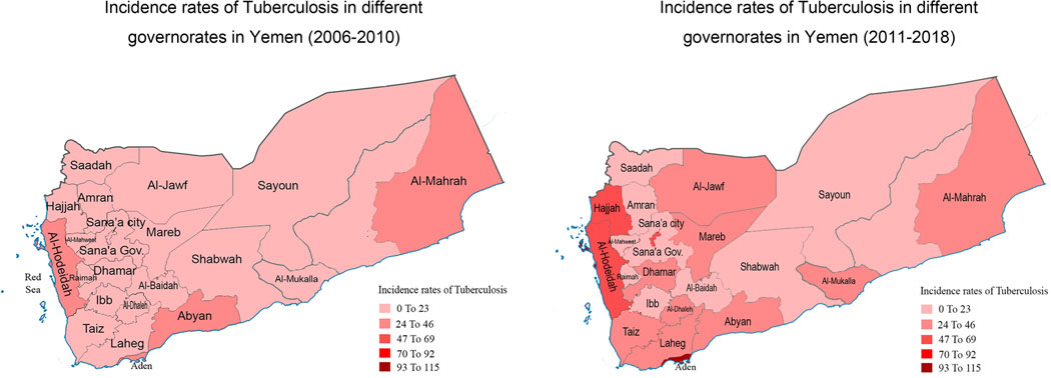
The incidence level of tuberculosis in different provinces (2006–2018).

When the comparison was made between the periods of 2006–2010 and 2011–2018 for the incidence of TB infection by governorates, there was a significant increase in TB infection in all governorates in the period 2011–2018 (Table 3). The highest increase occurred in Sana'a city, Sana'a Gov., Hajjah and Saadah with mean incidence increased by higher than two times in 2011–2018 than that in 2006–2010. For most other governorates, the incidence in 2011–2018 increased by approximately one time than that in 2006–2010 (Table 3, Fig. 4).

**Table 3.** The incidence of tuberculosis in different governorates from 2006 to 2018 in Yemen

Governorate	2006–2010			2011–2018			Incidence change^a^
	Average No. of cases	Mean population	Incidence	Average No. of cases	Mean population	Incidence	
Abyan	125	482 865	25.8	203	565 204	35.9	0.4
Aden	286	662 552	43.2	893	785 157	113.8	1.6
Al-Baidah	68	638 833	10.7	177	746 759	23.7	1.2
Al-Dhaleh	60	522 176	11.4	142	611 687	23.2	1
Al-Hodeidah	679	2 399 588	28.3	1377	2 816 698	48.9	0.7
Al-Jawf	87	489 616	17.7	171	571 630	29.9	0.7
Al-Mahrah	26	98 713	26.3	54	116 129	46.1	0.7
Al-Mahweet	51	548 479	9.2	116	641 880	18	1
Al-Mukalla	109	516 210	21.2	159	606 752	26.1	0.2
Amran	88	974 267	9.1	269	1 140 570	23.6	1.6
Dhamar	182	1 472 741	12.3	544	1 722 613	31.6	1.6
Hajjah	300	1 637 310	18.3	1081	1 915 632	56.4	2.1
Ibb	204	2 356 359	8.7	622	2 751 875	22.6	1.6
Laheg	161	802 603	20.1	367	940 235	39	0.9
Mareb	46	264 329	17.6	134	309 285	43.3	1.5
Raimah	33	436 389	7.7	55	510 068	10.7	0.4
Saadah	53	769 905	6.8	188	901 030	20.8	2
Sana'a city	335	1 960 093	17.1	1515	2 314 012	65.5	2.8
Sana'a Gov.	71	1 019 392	6.9	272	1 192 455	22.8	2.3
Sayoun	24	630 850	3.9	74	740 264	10	1.6
Shabwah	35	521 831	6.6	99	610 566	16.2	1.4
Taiz	490	2 652 087	18.5	802	3 100 409	25.9	0.4

a Fold change in incidence (cases/100 000 persons) from 2006–2010 to 2011–2018.

### Diseases associated with TB

During the 13 years, the most common concomitant diseases with TB are DM (14.0%), followed by brucellosis (6.1%), hepatitis (6.0%), rheumatoid arthritis (4.3%), renal disorders (2.5%) and HIV infection (2.5%). From 2006 to 2018, leukaemia and SLE occurred in only 97 and 12 patients, respectively (Table 4).

**Table 4.** Concomitant diseases with TB patients in Yemen from 2006 to 2018

Diseases	Number	Occurrence rate^a^
Diabetes mellitus	12 936	14
Brucellosis	5679	6.1
Hepatitis	5545	6
Rheumatoid arthritis	3966	4.3
HIV	2331	2.5
Renal disorders	2311	2.5
Leukaemia	97	0.1
SLE	12	0.01

a Occurrence rate present for the percentage of concomitant disease among all the tuberculosis patients (*N* = 92 482).

**Fig. 5.**
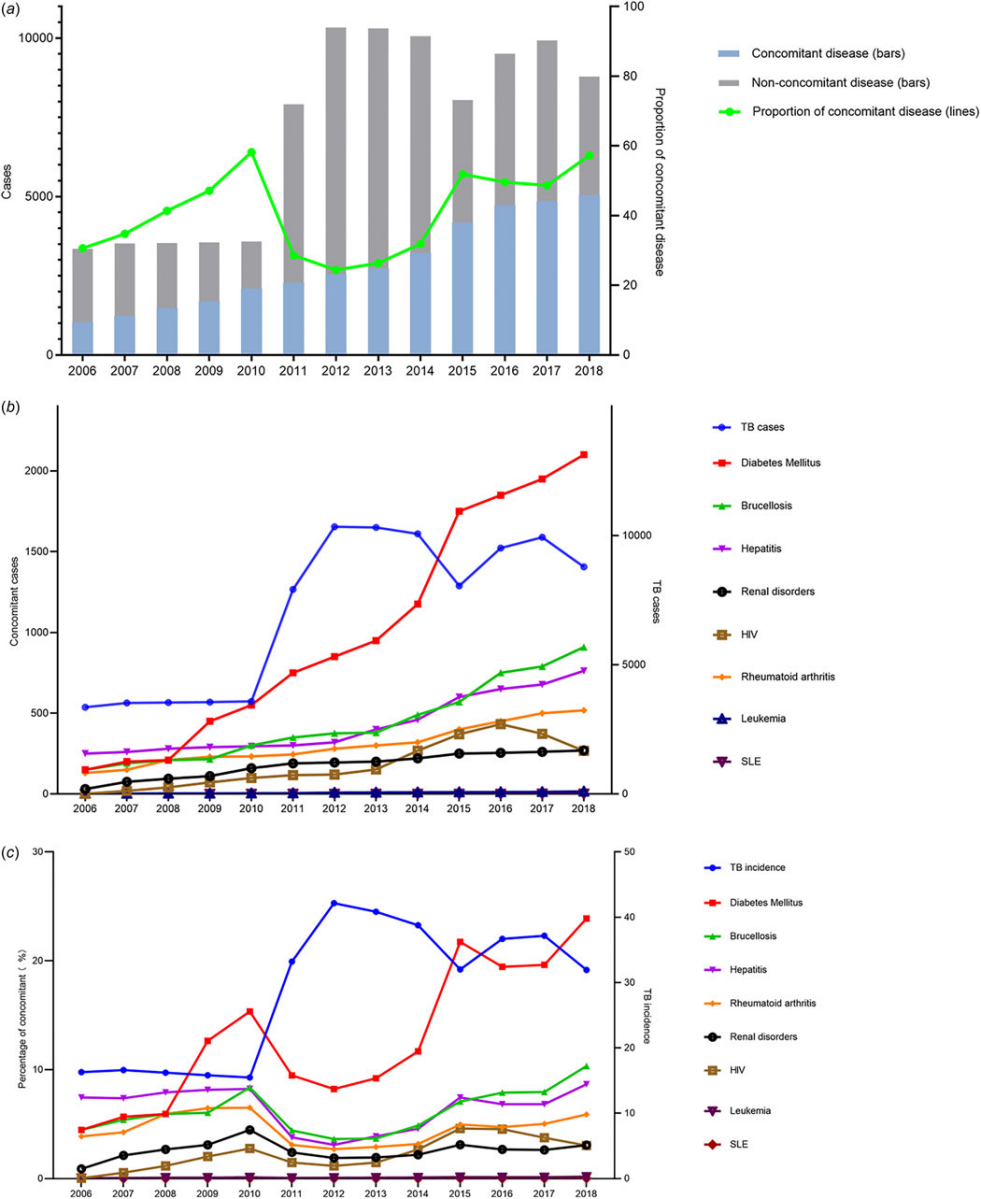
Comparison of incidence rates of concomitant diseases and total population. (a) The occurrence of the concomitant diseases each year. (b) Occurrence rate of the various concomitant diseases. (c) Comparison of proportion of various concomitant diseases and tuberculosis incidence.

From 2006 to 2018, the reported concomitant cases were increasing every year. Comparatively, the proportion of patients with concomitant diseases in all TB cases was decreased from 2010 to 2015 (Fig. 5a). Except for the sustained low level of SLE and leukaemia, the occurrence of other concomitant diseases was increasing by years, with DM rising the most (4.5–23.9%), followed by hepatitis (3.1–8.7%) and brucellosis (3.6–10.3%) (Figs 5b and 5c).

Nevertheless, the trends for occurrence rates of concomitant diseases are roughly the opposite to the trend for TB incidence rate. From 2011 to 2015, the incidence of TB was rising, while the proportion of each concomitant disease was declining during this period (Fig. 5c).

## Discussion

TB incidence in Yemen, according to the results of this study, ranged from 15.5/100 000/2010 to 42.2/100 000/2012 during the 13 years (Fig. 1). The incidence rates are similar to the National Population Survey (NPS) report that the country of Yemen bears an average burden of TB with the estimated number of incidents of National Strategic Program (NSP) (24/100 000 inhabitants) and 48 cases in all forms of TB. Our rate is lower than that in Africa (332/100 000) and Southeast Asia (278/100 000) but similar to those reported in the Middle East (36/100 000) [4]. It should be noted that this incidence may be an iceberg and the real rate is even higher, since many obstacles prevent patients in Yemen to access the TB centre for diagnosis and treatment. The incurring significant costs sometimes force the patients to abandon the diagnosis process and the following registration for TB treatment. However, there are no comprehensive studies of these barriers in Yemen, although the behaviour of health seekers has been documented elsewhere in the world.

The relapse TB in Yemen might be due to mismanagement including wrong diagnosis and delay of diagnosis, wrong or interrupted treatment, and the misuse of TB medicines, poor adherence to standardised treatment regimens, unregulated sale of anti-TB medicines and utilisation of TB medicines of unknown quality. In addition, there was difficulty for patients to reach centres for treatment due to the war situation in Yemen [5].

When comparing the TB incidence between the periods of 2006–2010 and 2011–2018, there was a significant increase in all governorates, all age groups in the period 2011–2018 (Table 3). How could the rise in incidence occur in the recent years? This may even happen over a short span of time. WHO and UNICEF are indicating that the reasons behind the rapid spread are high rates of malnutrition, food insecurity, collapsing health system, sanitation and clean water systems, which in turn due to the embroiled devastation in a regional conflict and Saudi-Emirati aggression. Over the past 6 years, key infrastructure in Yemen has been destroyed, which hampers the possibility of prevention and control of TB in the country [6]. The health system is struggling to cope, with more than half of all health facilities closed due to destruction or lack of funds. Furthermore, shortages in medicines and supplies are persistent and widespread. The main seaport to Yemen, Al-Hodeidah has been bombed and later on blocked for a period of time. The port is still partially blocked even after the fighting parties signed the Stockholm pace initiative at the end of 2018. This block disrupted the flow of the supplies of aid and other supplies into the country [7, 8].

Gender differences in TB, which mainly occur in people over 15 years old, have been reported in many settings, reflected by a male-to-female ratio of 1.69:1 globally [1, 9]. Nevertheless, the male predominance in TB incidence is region and time specific. A similar male and female incidence in Eastern Mediterranean Region (Yemen is among the counties of this WHO region) was demonstrated in the latest WHO Global Tuberculosis Report [1]. In our present study, male TB patients outnumbered the females in people over ages 15 during 2006–2010. In contrast, the gender disparities became unremarkable since the year of 2011. These findings are similar to the data in Yemen reported by WHO, in which the gender ratio was 1.3:1 in 2010 and 1.2:1 in 2011 [10, 11]. Therefore, we may postulate that the year of 2011 be the inflection point of the disappearance of gender differences in TB incidence. Gender-specific TB prevalence needs to be further investigated to ascertain the magnitude of sex difference, and to explore the social/cultural, and biological causes underlying the gender disparities through comparative studies in diverse settings from transmission of *M. tuberculosis* to successful recovery and rehabilitation.

TB in children under 15 years of age is also called paediatric TB, which account for 6–11% of all TB cases [1, 12]. We found that paediatric TB patients account for 9.6% in Yemen [12]. Our discovery is among the higher number of the contemporary global burden. Moreover, paediatric TB was increasing more rapidly during the period of 2011–2018 than other age groups. According to data released by WHO, national BCG immunisation coverage in Yemen ranged from 58% to 73% in 2006–2018 [13]. Considering low notification rate in children attributable to non-specific symptoms, uncertain diagnosis, poor access to health facilities, poverty and lack of awareness among families, it is estimated that only 50% of paediatric TB cases have been notified to national surveillance programmes [14]. Therefore, the real incidence for paediatric TB may surpass largely the notified numbers hereby.

UNICEF highlights that Yemen remains one of the largest humanitarian crises in the world, with around 20.7 million people including almost 11.3 million children (55% of the population) are now in need of humanitarian assistance [15]. Internal displacement and the dire hunger crisis exacerbated the pre-existed malnutrition. International aid organisations have been helping treat severe acute malnutrition in children by providing essential therapeutic food and medical supplies. On the ground of the TB prevention, WHO has launched a ‘Childhood TB Roadmap’ to improve case detection, diagnosis and treatment outcomes, and also calls for improved commitment and accountability at all levels of the health system and society [16]. In this context, activities for the detection of active cases include home screening, public–private mixing services, mobile chest health maps, community home care visits, screening of children in school as well as safe maternal health services [17].

When taking into account the diseases associated with TB, one may note that with the rapid growing of TB incidence from 2010 to 2015, the percentage of concomitant diseases was notably declining (Fig. 5c). This result demonstrated a sharp elevation of the TB and a slow and steady increase of the concomitant diseases. Among the combined diseases, DM, brucellosis, hepatitis and rheumatoid arthritis are most frequently found (Table 4).

Prevalence of DM is increasing worldwide, particularly in low- and middle-income countries. The pooled prevalence of DM in Eastern Mediterranean Region has over doubled since 1980, rising from 5.9% to 13.7% in the adult population [18]. A cross-sectional, population-based study in Yemen demonstrated that the overall prevalence of type II DM was 4.6% in adults aged 25–65 years [19]. DM can impair host immunity and lead to a threefold elevation in TB risk [20, 21]. Studies on the epidemiology of TB-DM showed particularly high prevalence rates of DM among TB in certain regions, including South India (54%), the Pacific Islands (40%) and northeastern Mexico (36%) [22–25]. Here we found that 4.5–23.9% of TB was combined with DM in Yemen during the period from 2006 to 2018, which is lower than the above regions. Similarly, a 5-year prospective study in 10 governorates of Yemen with participant recruited from 2007 showed the prevalence rate of DM among pulmonary TB was 8.1% [21]. However, Yemen is reported among the countries with both high prevalence of DM (7.7%) and TB (0.14%), based on a longitudinal analysis of 163 countries [26]. As the risk factor in TB infection and adverse treatment, the increasing numbers of patients with TB-DM comorbidity urge to implement strategies for TB prevention and control among the millions of DM patients exposed to *M. tuberculosis* in Yemen.

Apart from the DM, relatively high occurrence of brucellosis and hepatitis is characteristic for concomitant diseases in Yemen.

Brucellosis is a worldwide zoonotic disease infecting both animals and humans mainly by *Brucella abortus*, *B. mellitensis* or *B. suis*. It is transmitted to humans through contact with infected animals or their products. Brucellosis has been continuing to be an important burden in Yemen and other Middle East countries [27–29]. The recent available data in Yemen nationwide on notified incidence of human brucellosis are 88.6/100 000 in the year of 2016 [30]. Regional investigation of human brucellosis in Yemen showed varied seroprevalence, ranging from 0.3% in blood donors, 7.9% in general population, to 32.3% in slaughterhouse workers [31]. The infection risk factors of human brucellosis in Yemen have been attributed to social and political instabilities, lack of health care and disease control resources, livestock husbandry systems and traditional customs [30]. Drinking of raw milk could be thought as one of the unique risk factors, because people do drink raw milk daily without pasteurisation and in primitive methods that lack hygiene in many villages in Yemen. Brucellosis and TB are two chronic granulomatous infectious diseases that are endemic in the developing world. They resemble each other in the clinical pictures. The co-infection of TB and brucellosis is a very rare condition. Up to date there are two case reports from China involving two patients of spinal TB combined with brucellosis [32, 33]. In the present study, the notified brucellosis was diagnosed by the presence of clinical symptoms with positive serology for Brucella antibodies by the standard tube agglutination test. Information, including the site of TB lesion (pulmonary or extrapulmonary), age and gender, and residence location of patients combined with the brucellosis, has not been indicated in the archived data for the present study. However, considering the high burden of human brucellosis in Yemen, potential high frequency of the combined infection of the two diseases found in Yemen should be vigilant for surveillance and control.

Viral hepatitis is another major global health concern, with a prevalence of HBV or HCV from 1.5% to 10.8% in Yemen [34–36]. Chronic co-infections of hepatitis virus with TB were reported ranging from 0.5% to 44%, previously [37, 38]. Comparatively, the present study reveals a moderate rate of co-infections of the two diseases. Numerous studies have revealed age- and gender-specific disparities of viral hepatitis. But we regret that we do not possess data-related age and sex distribution of the TB patients co-infected with viral hepatitis. Considering the growing trend in the incidence of paediatric and female TB in Yemen, it is worth to explore whether there is an association between the viral hepatitis and the age- and gender-specific rising incidence of TB. The occurrence of both TB and concomitant hepatitis virus is associated with poor treatment outcomes. Therefore, targeted social assistance should be provided to such TB patients to improve clinical response to anti-TB therapy.

As for the coinfection of HIV with TB, the frequency is 2.5% (Table 4). Since the first case of HIV infection was detected in Yemen in 1987, the Ministry of Public Health and Population has established the National AIDS Program to monitor and control the spread of HIV. The main activities of the national action programme included providing antiretroviral treatment for people living with HIV in five governorates, providing voluntary counselling and testing services in 36 locations in several governorates, and preventing mother-to-child transmission. In 2004, the programme followed the Directorate of Primary Health Care. The programme receives reports every 3 months from different locations of the NAP. According to the 2011 HIV estimates of the National Action Program, the HIV prevalence rate in Yemen was low (0.2%) [39]. A total of 3995 HIV cases were reported during the years 1987–2013. HIV/AIDS is not a leading co-infection with TB in Yemen. The Eastern Mediterranean region, including Yemen, had consistently low rate of HIV/AIDS (3–0.99%) from 2005 to 2018. The reason for the lower prevalence of HIV might be attributed to some custom among Muslim populations which contribute to the decreased risk of HIV transmission. One is low alcohol use, which reduces disinhibition and hence the risky behaviour. Another is male circumcision, which was shown to reduce infection in a recent trial [40].

The war in Yemen has led to the emergence of one of the worst humanitarian crises in the world. With weak health systems, water and sanitation infrastructure, and a collapsing economy, diseases and epidemics spread throughout the communities. Our data demonstrated a high probability of occurrence of TB in Aden, Al-Hodeidah and Hajjah, and the incidence increase more than doubled in Sana'a city, Sana'a Gov., Hajjah and Saadah.

Sana'a is the official capital city of Yemen. Aden is the chief port and the former economic centre. Hajjah is located northwest of Sana'a, due north of Al-Hodeidah. Al-Hodeidah, a city in western Yemen, is another chief port. Al-Hodeidah, Hajjah and Aden are poverty areas than Sana'a Gov. Al Hodeidah and Sana'a Gov. have the most population density. Al Hodeidah and Hajjah have the worst social economic conditions. Saadah is an agriculture area located in the far north of Yemen. These districts, all with high population density, have been affected most by the war, which may account for their higher prevalence.

This study has some limitations. One may design cross-section only for time links. The health facility-based study is another limitation that may not allow generalisations about the exact situation in the community. However, given the limited resources available in Yemen, alternative ways of exploring TB in society are not possible. The sample size may also be considered insufficient and the information collected is a limitation of this study; however, this was the total number of cases and information in which the control programme was notified during the study period for 13 years. Despite these limitations, we assume that this study is the first of its kind in Yemen, reflecting the epidemiology of TB, with a special focus on the sex, age, localities and years of TB indicators. Our findings support the development of interventions that address increase incidence of TB in children and concomitant diseases by other infectious agents during persistent unrest.

## Conclusion

We conclude from this study that TB is prevalent in Yemen, with a significant increase in the incidence after the year 2011. A high and rapid rising incidence of TB in children was observed. Sana'a city, Hajjah, Sana'a Gov. and Saadah have undergone dramatically increased incidence. Except for DM, characteristic concomitant disease with TB is brucellosis and hepatitis. Development of interventions to reduce TB incidence in children and concomitant communicable diseases during persistent unrest is urgently needed.

## Data Availability

All data are publicly available and listed in this article.
