# Prevalence and surveillance of tuberculosis in Yemen from 2006 to 2018 – ERRATUM

**DOI:** 10.1017/S0950268822001583

**Published:** 2022-10-28

**Authors:** Wadee Abdullah Al-Shehari, Yi-An Yin, Xinyang Wang, Ying Wang, Haobo Sun, Yingmei Fu, Fengmin Zhang

**Affiliations:** 1 Department of Microbiology, Basic Medical Sciences College, Harbin Medical University, Harbin 150086, China; 2 Department of Medical Microbiology, Faculty of Sciences, Ibb University, Ibb, Yemen; 3 Wu Lien-Teh Institute, Harbin Medical University, Harbin 150086, China

The original paper was published with one (instead of three) corresponding author – Dr Yingmei Fu – who remains a corresponding author. Two more scholars were omitted as correspondent authors for the article above. It should have been published with the following scholars included:

**Prof. Zhang Feng-Min** Harbin Medical University, China Email Address: fengminzhang@ems.hrbmu.edu.cn

**Wadee Abdullah Alshehari** Ibb University, Yemen Email Address: dr.wadee40@yahoo.com

Both the article's PDF and HTML have been updated to reflect this change. The publisher apologizes for the omission.
